# Hypercholesterolemia and microvascular dysfunction: interventional strategies

**DOI:** 10.1186/1476-9255-7-54

**Published:** 2010-11-18

**Authors:** Phoebe A Stapleton, Adam G Goodwill, Milinda E James, Robert W Brock, Jefferson C Frisbee

**Affiliations:** 1 Center for Cardiovascular and Respiratory Sciences, West Virginia University School of Medicine, 1 Medical Center Drive, Morgantown, WV 26506, USA; 2 Division of Exercise Physiology, West Virginia University School of Medicine, 1 Medical Center Drive, Morgantown, WV 26506, USA; 3 Department of Physiology and Pharmacology, West Virginia University School of Medicine, 1 Medical Center Drive, Morgantown, WV 26506, USA

## Abstract

Hypercholesterolemia is defined as excessively high plasma cholesterol levels, and is a strong risk factor for many negative cardiovascular events. Total cholesterol levels above 200 mg/dl have repeatedly been correlated as an independent risk factor for development of peripheral vascular (PVD) and coronary artery disease (CAD), and considerable attention has been directed toward evaluating mechanisms by which hypercholesterolemia may impact vascular outcomes; these include both results of direct cholesterol lowering therapies and alternative interventions for improving vascular function. With specific relevance to the microcirculation, it has been clearly demonstrated that evolution of hypercholesterolemia is associated with endothelial cell dysfunction, a near-complete abrogation in vascular nitric oxide bioavailability, elevated oxidant stress, and the creation of a strongly pro-inflammatory condition; symptoms which can culminate in profound impairments/alterations to vascular reactivity. Effective interventional treatments can be challenging as certain genetic risk factors simply cannot be ignored. However, some hypercholesterolemia treatment options that have become widely used, including pharmaceutical therapies which can decrease circulating cholesterol by preventing either its formation in the liver or its absorption in the intestine, also have pleiotropic effects with can directly improve peripheral vascular outcomes. While physical activity is known to decrease PVD/CAD risk factors, including obesity, psychological stress, impaired glycemic control, and hypertension, this will also increase circulating levels of high density lipoprotein and improving both cardiac and vascular function. This review will provide an overview of the mechanistic consequences of the predominant pharmaceutical interventions and chronic exercise to treat hypercholesterolemia through their impacts on chronic sub-acute inflammation, oxidative stress, and microvascular structure/function relationships.

## Introduction

While hypercholesterolemia, defined as excessively high plasma cholesterol levels, has emerged as a strong risk factor for cardiovascular disease (CVD). Data acquired by the National Health and Nutrition Examination Survey (NHANES) 2005-2006 found that the mean total serum cholesterol for Americans over the age of 20 was 199 mg/dl, approximating the American Heart Association (AHA) recommended level of 200 mg/dl [1]. Unfortunately, 16% of adults were found to have total cholesterol levels of more than 240 mg/dl, a level considered by the AHA to carry twice the CVD risk of those individuals at the desired level [1,2].

Total cholesterol can be broken down into a diagnostic lipoprotein profile, including high density lipoprotein (HDL), low density lipoprotein (LDL), intermediate density lipoproteins (IDL), very low density lipoprotein (VLDL), chylomicron remnants, and triglycerides. With respect to these markers, the AHA publishes recommendations summarized in Table 1[1]. HDL is considered to be beneficial as higher levels have been correlated with reduced risk of negative cardiovascular events, in large measure by promoting reverse cholesterol transport, an anti-atherogenic process resulting in cholesterol from peripheral tissues returning to the liver for subsequent processing [1]. Elevated LDL cholesterol and triglycerides are considered detrimental as their increased concentration is well correlated with poor cardiovascular outcomes [1,3]. Ongoing study has also suggested that IDL, VLDL, and chylomicron remnants may also play an active role in peripheral vascular (PVD) and coronary artery disease (CAD) development [3].

**Table 1:** American Heart Association guidelines for cholesterol and triglycerides levels in adults. Last updated 7/2/09

	Total	LDL	HDL	Triglycerides
Optimal	-	< 100 mg/dL*	> 60 mg/dL	-

Near optimal/above optimal	< 200 mg/dL	100 - 129 mg/dL	40-50 mg/dL (men) 50-60 mg/dL (women)	< 150 mg/dL

Borderline high	200-239 mg/dL	130 - 159 mg/dL	-	150-199 mg/dL

High	-	160 - 189 mg/dL	-	200-499 mg/dL

Very high	≥ 240 mg/dL	> 190 mg/dL	< 40 mg/dL (men) < 50 mg/dL (women)	≥ 500 mg/dL

* If the patient has additional risk factors LDL levels are recommended under 70 mg/dL.

As high total cholesterol levels are considered to be a major independent risk factor for development of PVD and CAD, considerable attention has been directed toward evaluating the impact and mechanisms of cholesterol lowering therapies and interventions for cardiovascular outcomes [2-4]. Cholesterol has been shown to interrupt and alter vascular structure and function as it builds within the lining of the vascular wall, and can interfere with endothelial function leading to lesions, plaques, occlusion, and emboli; along with a reduction in healing, recovery, and appropriate management of ischemia/reperfusion injury [5-9]. With specific relevance to the microcirculation, it has been clearly demonstrated that evolution of hypercholesterolemia is associated with endothelial cell dysfunction [5,10-14]. Additionally, reports have shown a near-complete abrogation in vascular nitric oxide (NO) bioavailability, elevated oxidant stress, and the creation of a strongly pro-inflammatory condition; symptoms which can culminate in profound impairments to vascular reactivity [10,12,15-22]. Investigation into vascular consequences of chronic hypercholesterolemia, the mechanisms through which these consequences occur, and the potentially beneficial effects of ameliorative therapies have received considerable attention in recent years [3,9,12,15,17,23-26].

Although a substantial risk factor for CVD, hypercholesterolemia has also been demonstrated to be manageable, as summarized in meta-analytic projects which have supported the use of pharmaceutical interventions to reduce cholesterol, with the outcome of lowering cardiovascular event incidence [24,27]. However, effective interventional treatment can be problematic, as the presence of specific genetic risk factors are frequently present. The condition of familial hypercholesterolemia (FH) is an inherited autosomal dominant disorder caused by variations to the low density lipoprotein receptor (LDLR) gene, preventing effective function and dramatically elevating levels of circulating LDL [28]. While the phenotypic effects of the homozygous condition are more severe, the prevalence of the heterozygous condition affects approximately 1 in 500 individuals [29]. Normally, LDL transports cholesterols and fats through the aqueous bloodstream to the cell surface where LDLR mediates its endocytosis, a process that is rendered ineffective in FH. A second inherited cause of hypercholesterolemia is familial combined hyperlipidemia (FCH), also known as type III hyperlipidemia, which presents high cholesterol and high triglyceride levels stemming from a number of gene polymorphisms [30]. Interestingly, while the dyslipidemic profile of these two conditions differs, there is a striking similarity in the poor vascular outcomes [8,12].

## Hypercholesterolemia and Vascular Dysfunction

The vascular endothelium, a single cell layer on the inner surface of all vessels, is capable of producing numerous bioactive molecules, thereby acting as an autocrine, paracrine, and endocrine organ [26]. In a normal system, endothelial cells maintain vascular tone via endothelium-derived relaxing factors including NO, prostacyclin, and endothelium-derived hyperpolarizing factors [14] in an integrated balance with sympathetic and myogenic tone as well as parenchymal cell influences. These molecules help to regulate the homeostasis of the vascular system by adjusting to a number of systemic demands on blood flow, coagulation, inflammation, platelet aggregation, and signal transduction, with any decay in efficacy considered as dysfunction [31].

Nitric oxide (NO), a gas synthesized from the amino acid L-arginine through the enzyme nitric oxide synthase (NOS), has been widely considered as an endothelium-dependent regulator of vascular tone, with additional roles in preventing platelet activation, inhibiting oxidative stress, cell growth, and inflammation, among others [16,32]. Asymmetric dimethylarginine (ADMA) is an endogenous inhibitor of NOS through competition with L-arginine [22]. Given recent studies demonstrating an increased endogenous production of ADMA in hypercholesterolemia and the inverse relationship between NO production and ADMA concentration, ADMA elevations are currently under intensive evaluation as an additional risk factor for CVD [20,22].

Previous studies within our laboratory and others have shown that dilator reactivity in response to NO-dependent stimuli is moderately impaired in hypercholesterolemic mice as compared to responses in control animals [12,19,33-37]. This reduction is not due to an inability to react to the NO signal, as vessels are able to respond normally to NO donors, rather there is a reduction in the bioavailability of NO within vasculature either via deficits in production or due to increased oxidant scavenging [13]. Additional data suggests that NO-mediated endothelium-dependent responses within a hypercholesterolemic milieu may differ between conduit vessels and the microcirculation, as peripheral resistance arterioles have a greater sensitivity to local metabolite production [38-40]. Further, in hypercholesterolemic mice and diet induced hypercholesterolemic rabbits, compensatory mechanisms evolve to maintain endothelium-dependent dilation as a result of a decrease in NO bioavailability, and appear to involve altered patterns of arachidonic acid metabolism involving both the cyclooxygenase and lipoxygenase pathways [11,12,23,41-43]. Arachidonic acid action within hypercholesterolemia is not limited to metabolite production inducing dilation, but includes the production of thromboxane A_2 _(TXA_2_), a potent vasoconstrictor [15,44]. Hypercholesterolemic animals have shown a limitation to arachidonic acid induced dilation due to an increase in TXA_2 _production during metabolism [15]. Similar hypercholesterolemic animals have shown an improvement in vascular reactivity and atherosclerotic lesions in animals who are thromboxane receptor deficient [15,45,46].

The vascular consequences of lipoprotein remnants within the hypercholesterolemia, independent of but in addition to endothelial dysfunction, can lead to organ dysfunction and subsequently greater systemic consequences due to an impairment of tissue perfusion. This impairment can be classified as arteriolar remodeling or capillary rarefaction due to the buildup of cholesterol within the hyperlipidemic population. Rarefaction may play a role in many of the systemic effects stemming from structural pathologies reported within this population, including but not limited to changes within the skin, glomerulopathy leading toward kidney dysfunction and hypertension, reductions in coronary flow reserve leading to an early coronary heart disease and hepatic dysfunction leading toward non-alcoholic fatty liver disease [47-51].

## Hypercholesterolemia and Inflammation

**Figure 1:**
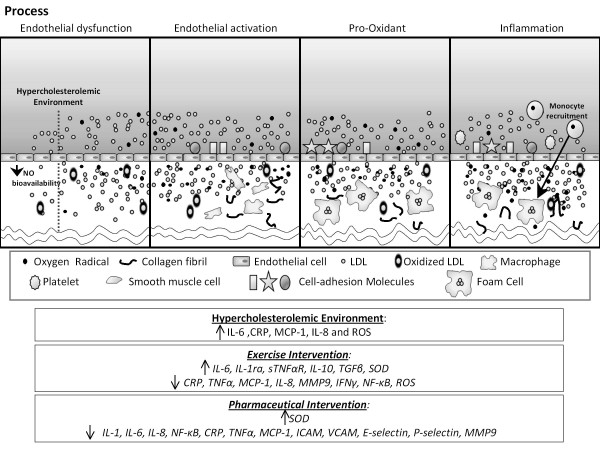
**Figure illustrates the vascular progression of disease within a hypercholesterolemic environment**. The depiction gives a simplified version of the process, while including documented signaling adaptations associated with hypercholesterolemia, pharmaceutical therapies, and exercise interventions

Numerous studies have clearly established that hypercholesterolemia leads to an inflammatory response within the microvasculature, reflected by endothelial cell activation, leukocyte recruitment, rolling and adherence, as well as platelet activation and adhesion characterized in Figure 1[18,52]. Platelet activation can initiate leukocyte recruitment to lesion prone areas as evidenced by an increased surface CD40 expression indicative of cellular activation [18,53]. Leukocyte activation can subsequently obstruct capillary networks, reducing capillary perfusion - a condition previously identified in hypercholesterolemia [19].

The decreased bioavailability of NO in hypercholesterolemia also diminishes the anti-inflammatory properties of the endothelial cell, permitting the activity of growth factors on the cell surface and platelet activation to act as chemoattractants to a parade of inflammatory events. Leukocytes begin to roll along the lumen and adhere to the cell wall, extravasating due to an increase in vascular permeability, and residing within the intimal space [22]. Monocyte chemotactic protein-1 (MCP-1) and interleukin-8 (IL-8) have both been found to be important in hypercholesterolemic patients, acting to increase monocyte recruitment and adherence which leads to wall remodeling [6,54-56]. Macrophages, derived from monocytes, begin to accumulate LDL and oxidized LDL (oxLDL) which develop into foam cells between the basal lamina of the endothelium and the smooth muscle layer [26]. These foam cells lead to the production of numerous inflammatory and oxidative stress markers, cytokines, chemokines, and growth factors which aggravate the balance of endothelial equilibrium leading to vascular dysfunction [57].

Elevated cholesterol has also been shown to trigger the release of the inflammatory mediator C-reactive protein (CRP), a useful clinical marker of CVD [58,59]. It is hypothesized that CRP, via IL-6, may exacerbate vascular dysfunction by inhibiting eNOS, stimulating production of reactive oxygen species and increasing vascular permeability, and may also initiate the expression and stimulation of adhesion molecules, chemokine production, and thrombus formation within endothelial cells [54]. Unfortunately, as a cellular marker of vascular inflammation, the source of CRP within the hypercholesterolemic condition is unclear [60].

## Hypercholesterolemia and Oxidative Stress

Excess oxidative stress is caused by an imbalance between pro- and anti-oxidant enzymes, leading to an overproduction of free radicals, including superoxide, hydroxyl radicals, and lipid radicals, which may damage cellular components, interfering with normal function again characterized in Figure 1. Other molecules such as peroxynitrite, hydrogen peroxide, and hypochlorous acid are also oxidants, but are not free radicals. The two major sources of oxidants within the vasculature are leukocytes (macrophages) recruited due to an endothelial injury signal and inefficiencies within smooth muscle cell mitochondrial metabolism [61].

Hypercholesterolemia may also increase activity of three major oxidant producing enzyme systems; NADPH oxidases (NOX), xanthine oxidase, and myeloperoxidase. NOX acts to transfer an electron to an oxygen molecule, forming superoxide ultimately H_2_O_2 _[62]. While seven NOX isoforms have been identified (NOX1-5, DUOX 1 and 2), four of these (NOX1, 2, 4, and 5) have been recognized within the vascular wall, with NOX2 responsible for the greatest impact on ROS-related decreases to NO bioavailability [63]. Xanthine oxidase forms superoxide and H_2_O_2 _during the reduction of oxygen, while myeloperoxidase is produced by neutrophils and monocytes and produces a toxic hypochlorous acid; within a pathological condition overactive enzymes can lead to the overproduction these radicals, leading to scavenging of NO molecules, uncoupling of eNOS, and/or the formation of peroxynitrite [61]. eNOS uncoupling and substrate reduction (tetrahydrobiopterin (BH_4_) and L-arginine), can transform eNOS into a superoxide generating enzyme which can, in turn, produce greater amounts of oxidant radicals and hydrogen peroxide in addition to NO production [32,64].

A range of antioxidant mechanisms are in place to minimize and balance the effects of ROS, including superoxide dismutase (SOD), glutathione peroxidase (GPx4), catalase, and thioredoxin reductase. SOD, which comes in three forms, soluble cytoplasmic (SOD1), extracellular (SOD3) containing copper and zinc and mitochondrial (SOD2) containing manganese, is the main cellular antioxidant system in all cell types and is capable of converting superoxide radicals to H_2_O_2 _and oxygen [58,65,65]. GPx4 reduces H_2_O_2 _and lipid peroxides to water and lipid alcohols, and reduces the development of atherosclerosis during hypercholesterolemia through the inhibition of lipid peroxidation and a decreased sensitivity of endothelial cells to oxidized lipids [66]. Catalase acts to reduce hydrogen peroxide to oxygen molecules and water. Within the pathological state of hypercholesterolemia, antioxidant systems are unable to handle the increased demand and the ROS production exceeds capacity.

Within hypercholesterolemia, reactions between oxygen radicals or enzymatic oxidation and lipoproteins or more specifically phospholipids can lead to production of lipid radicals (oxLDL) or oxidized phospholipids (OxPL). These OxPL can interact with membrane receptors to accumulate within the cellular membrane, disrupting normal cellular function through a reduced bioavailability of NO, eliciting an immune response, leading to poor vascular function, and ultimately atherosclerosis [14,67,68]. This conclusion is further supported with evidence that cholesterol fed animals with polyethylene-glycolated-SOD demonstrate an improved endothelium dependent dilation, while normocholesterolemic animals did not show any effects [69]. OxPL can interact directly with the endothelial cell through interactions with the lectin-like oxLDL receptor (LOX-1), an endothelial receptor for oxidized LDL in endothelial cells; this receptor is induced by a variety of inflammatory cytokines, oxidative stress, hemodynamic changes, and abundance of ox-LDL [70]. In addition to oxLDL, LOX-1 can bind advanced glycation end products (AGE), activated platelets, and leukocytes all furthering inflammatory and oxidative processes [70]. Lastly, as the interactions with oxPL cause further injury subsequently activating the endothelial cell and platelets, signaling a variety of adhesion and inflammatory molecules including MCP-1, leading to monocyte recruitment, diapedesis, macrophage differentiation, and foam cell formation only further aggravates the delicate system by producing additional ROS and inflammatory recruitment [68,71].

## Hypercholesterolemia and Pharmaceutical Therapies

Statins, 3-hydroxy-3-methylglutaryl-coenzyme A (HMG CoA) reductase inhibitors, are currently one of the most widely prescribed drugs on the market. They target liver HMG CoA reductase activity and inhibit the production of a cholesterol precursor, mevalonic acid. They also specifically act to change the conformation of HMG-CoA reductase when bound, preventing a functional structure [25]. This enzymatic inhibition acts to prevent protease activation of sterol regulatory element binding proteins (SREBPs) from the endoplasmic reticulum, thereby preventing nuclear translocation and upregulation of LDL gene expression, limiting hepatic cholesterol production [25].

Statins have been identified to have numerous positive outcomes associated with their direct cholesterol lowering [72-75]. However, in addition to these, vasculoprotective properties such as increased NO bioavailability, antioxidant, anti-inflammatory and immunomodulatory properties leading to an overall improvement of endothelial function have also been identified; yet specifically identifying the discrete result in human hypercholesterolemic patients is difficult as the cholesterol lowering benefits are similar [76,77]. Additionally, statin therapy has been found to significantly improve endothelial function (based on flow-mediated dilator responses) in hypercholesterolemic patients who had also been diagnosed with peripheral artery disease [78]. While this beneficial effect may have resulted from an increased NO bioavailability, the underlying mechanisms have not been fully understood [79].

These diverse positive vascular outcomes are most easily identified while using a genetically modified murine model, as the lipid-lowering results become null, leaving the pleiotropic effects evident. While similar to the secondary benefits of direct cholesterol lowering, these independent effects described include: reducing inflammation, decreases in ROS, increases in NO bioavailability and endothelial function, decreases in platelet activation and aggregation, reduction in coagulation and decreases in cellular proliferation, among others [42]. Unfortunately, at this time while the independent outcomes are evident, the mechanisms of action leading to these improvements are not fully elucidated.

Ezetimibe (Zetia) is a selective agent which acts to prevent cholesterol absorption in the intestine through targeting Niemann-Pick C1-like 1 protein (NPC1L1), which is expressed on the intestinal cell surface and is a transporter with secretion signal and sterol-sensing domains. Ezetimibe will inhibit this protein, thereby blocking LDL uptake from the intestine [80]. The subsequent reduction in cholesterol transport to the liver stimulates a compensatory increase in LDLR expression, thereby increasing vascular clearance with no known serious side effects [9]. While cholesterol lowering therapies have shown a positive correlation with reductions in cardiovascular events, ezetimibe has recently begun to show pleiotropic effects such as reductions in liver lipids, reductions in lipid lesions, reductions in ADMA levels, and increases in eNOS mRNA expression [26,75].

When used in combination, ezetimibe and statins (e.g., Vytorin) act via complementary pathways to prevent cholesterol absorption from the intestine and hepatic production. Long term co-administration of these drugs have been shown to reduce LDL blood cholesterol levels by 60% while concurrently raising HDL levels and limiting liver toxicity, myotoxicity and/or rhabdomyolysis traditionally caused by statin treatment alone [9,81,82]. However, at present, the side effects of the combined therapy are not well described, and it is unclear how effective these are for impacting the inflammatory profile [73,74,83].

## Oxidant Stress, Inflammation and Pharmaceutical Therapies

While lowering overall cholesterol levels can lead to a decrease in vascular oxidative stress and thereby improve endothelial function, some groups have found antioxidant properties to be a pleiotropic effect of statins [84]. When evaluated to examine NO in a biologically active form, cholesterol lowering drugs were shown to increase the efficiency of the NOS system, while simultaneously showing an inactivation of oxygen radicals within the system [85]. These drugs may not act directly upon the radicals, but instead act to reduce oxidant stress by decreasing substrate availability for these radicals to act upon or by increasing antioxidant enzymatic activities, such as SOD [86]. Statins have found to act upon the p21 Rac protein interrupting the NOX subunit assembly working directly to inhibit the production mechanism of superoxides through disruption of the NOX enzyme [87]. Some studies have shown positive results with respect to lipid peroxidation, including the increase of an antioxidant effect leading to a decrease in ox-LDL with combination ezetimibe/statin treatment [88].

Pharmaceutical treatments have been shown to influence inflammation through the decrease of systemic markers of inflammation and to increase the stability of existing plaques, thereby reducing the risk for thrombosis. Some groups are considering treating LDL as means to managing inflammation and preventing atherosclerotic lesions with mixed reviews and results [89,90].

CRP has been commonly used as a marker of inflammation in a clinical setting since it is associated with low-grade cardiovascular inflammation. Statin drugs have been shown to decrease CRP in numerous human studies, including JUPITER, ENHANCE, CARE, and PRINCE, regardless of their lipid lowering effects [91]. Additional studies have shown an interference with the inflammatory process, impacting the expression of interleukins, adhesion molecules, platelet aggregation, and chemoattractants including IL-1, IL-6, IL-8, NF-κB, and TNF-α culminating in the decrease of CRP [92].

Animal studies have shown atorvastatin to reduce inflammatory markers such as MCP-1 and the activation of the nuclear factor NF-κB [93]. More recently, as the pleiotropic effects of these interventions are being evaluated, some studies have found reductions in the adhesion molecules ICAM, VCAM, E-selectin, P-selectin, and platelet aggregation. These reductions are leading some to the conclusion that pharmaceutical therapies may reduce or limit the formation and instability of atherosclerotic plaques [94].

## Hypercholesterolemia and Exercise

The AHA and American College of Sports Medicine (ACSM) have recently released joint guidelines recommending aerobic and resistance physical activities for individuals under the age of 65 to maintain health, reduce risk of chronic disease states, and manage current risk factors including hypercholesterolemia [95-97]. Hypercholesterolemia has been shown to impair aerobic capacity by impairing dilator regulation, thought to be due to a lack of vascular reactivity stemming from a reduction in NO bioavailability [98]. However, this decline in vascular reactivity may also be due to wall remodeling as seen in the LDLR mouse model of FH or poor blood flow distribution due to microvessel rarefaction seen in the ApoE mouse model of FCH [56]. These may lead to a decrease in oxygen transport to working skeletal muscles during the hyperemic demand of exercise, further reducing aerobic capacity [98].

Few groups examine dose-response relationships between exercise training and cholesterol adaptations. Some have suggested that exercise can alter blood lipids at low training volumes, although effects may not be significant until certain caloric thresholds are met. Exercise training has rarely been shown to have a direct effect on total cholesterol or LDL levels; however, significant increases in HDL and decreases in triglycerides have been identified [99]. This may be a function of activity intensity, as a 1200 - 2200 kcal/week exercise program performed at moderate intensities, has been shown to reduce total and LDL cholesterol levels [99].

A number of moderate-intensity exercise programs have shown improvements to systemic aerobic capacity, effectively reversing early stage hypercholesterolemic changes within the vasculature, including improved vascular reactivity, NO bioavailability and eNOS activity [40,100]. These increases in NO bioavailability in humans and animal models of hypercholesterolemia have been attributed to eNOS expression and production of NO, due to a chronic rise in shear stress with exercise, as opposed to an increase in SOD or reduction in oxidant stress [101]. Exercise and shear stress have also been shown to improve mechanisms of endothelial vasodilation other than NO, such as prostaglandin release [12]. Exercise has also been shown to ameliorate increases in inflammatory and oxidative stress markers during chronic disease state, which would benefit many low-grade inflammatory conditions [102].

## Inflammation, Oxidant Stress and Exercise

In the past, inflammation associated with physical activity has been described as the reaction to a number of repeated micro-traumas to the muscle [103]. However, muscle has recently been identified as an endocrine organ, possessing the ability to manufacture and release humoral mediators directly into the system in response to muscle contraction [104]. This establishes a link between skeletal muscle activity and anti-inflammatory effects [105]. The cytokines produced, identified as myokines, include IL-6, IL-8, IL-15, brain-derived neurotrophic factor (BDNF), leukemia inhibitory factor (LIF) FGF21 and follistatin-like-1: each are regulated in some manner by the contraction or contractility of muscle [106]. With respect to IL-6, the myokine hypothesis suggests that both type I and type II muscle fibers are capable of producing and releasing IL-6, which may act locally through AMPK signaling or systemically to improve hepatic glucose production and lipid metabolism [107].

During acute exercise, there is an immediate increase in a variety of anti-inflammatory cytokines, such as IL-6, IL-1ra, sTNFR (soluble TNF-α receptor), and IL-10. However, pro-inflammatory cytokines TNF-α (tumor necrosis factor-α) and IL-1 are generally not changed [108]. Chronic exercise leads to a reduction of systemic and local markers of inflammation within the vasculature has been well established within the literature [109]. As exercise persists to a chronic state pro-inflammatory markers CRP, TNF-α, IFN-γ, MCP-1, IL-6, IL-8, and MMP-9 have all been shown to decrease from initial baseline levels; whereas anti-inflammatory markers IL-10 and TGF-β increase indicating the development of a less inflammatory phenotype [110,111]. The timeline and exact mechanisms by which a chronic increase in activity will lead to modest improvements in low-grade inflammation are uncertain [112]. However, some groups are focusing on the "long-term anti-inflammatory effects of exercise" [105,110].

Cellular respiration and metabolism are directly linked to physical activity and exercise as they are the source responsible for muscle action. In the presence of oxygen, aerobic respiration allows for the production of ATP, where glucose is broken down to pyruvate and enters the mitochondria for further processing via Kreb's cycle and oxidation via the electron transport chain. Unfortunately, minor inefficiencies within the mitochondria, including leaky membranes and limited cofactor availability, lead to a reduced ATP generation and the excess buildup of oxidants [113].

In acute exercise alterations to the mitochondrial electron transport chain is a direct source of oxidant stress due to the significant amount of oxidative handling throughout the system [92]. Therefore, any inefficiencies associated within this system are multiplied as mitochondrial requirements increase due to an increase in activity, specifically during acute exercise when there is an increase in whole body oxygen consumption thereby increasing the generation of ROS by active tissues [114]. During the production of these mitochondrial-derived radicals, there is also an increase of the pro-oxidant enzymes xanthine oxidase, myeloperoxidase, and NOX [115]. The upregulation of these enzymes causes an increase in plasma markers of ROS, such as F_2_-isoprostanes [116]. This increased oxidant stress, while promoting negative cardiovascular effects, has recently been shown to occur in conjunction with increases in antibodies to ox-LDL and antioxidant enzymes (catalase) after one week of activity in mice [117]. These changes suggest that after only a week of moderate activity, there is an initiation to improve hypercholesterolemia, limit the progression of foam cell development, and increase antioxidant enzyme activity within exercising and sedentary states. As exercise persists, mitochondrial and antioxidant enzymes also improve; specifically, an increase in expression of Cu/Zn superoxide dismutase (SOD-1) and glutathione peroxidase lead to a higher oxidant handling capacity and contribution to improved function [101,118]. As a consequence, there is a decrease in the plasma markers of oxidative stress F2-isoprostane, myeloperoxidase, and malondialdehyde [119]. Exercise training has also been shown to have a direct positive effect on the induction of eNOS and ecSOD (endothelial cell SOD), potent antioxidants. These increases are interdependent, as eNOS^-/- ^mice seem to be unaffected an increase in ecSOD [120].

Exercise and increases in NO have also been shown to induce HO-1 (heme oxygenase-1) expression. HO-1 products have similar anti-oxidant and anti-inflammatory effects, in addition to the inhibition of NF-KB an oxidant stress sensitive transcription factor [121]. The inhibition of NF-kB leads to a decrease of the entire downstream signaling cascade, which could be the link to many of the NO-mediated anti-inflammatory effects observed with chronic exercise such as decreases in leukocyte binding, chemotaxis, aggregation of platelets, and proliferation of smooth muscle cells [122].

## Conclusion

Given the severity of hypercholesterolemia as a risk factor for the progression of negative CVD outcomes, the pathways of effective interventional strategies to manage cholesterol levels, improve vascular reactivity, and restore NO bioavailability warrant continued investment. Pharmaceutical therapies have presented a variety of vasculoprotective effects which are not fully understood, but involve a complex interaction between vascular signaling mechanisms, oxidant stress and chronic inflammation. Additionally, physical activity and exercise have long been suggested as means to modify CVD and manage cholesterol. Current evidence also supports the theory of a long term anti-inflammatory effects through modifications of the IL-6 and CRP pathways, along with anti-oxidative effects of increased anti-oxidant enzyme expression and activity leading to a higher oxidant handling capacity at rest and during activity. These data suggest that the pleiotropic effects of exercise and conventional pharmaceutical therapies may be most beneficial when used in combination.

## Competing interests

The authors declare that they have no competing interests.

## Authors' contributions

PS conceived of the review, performed the literature search, compiled, designed, and drafted the manuscript. AG aided in the literature search and drafted the manuscript. MJ aided the literature search. RB conceived of the review, participated in the design, and execution. JF conceived of the review, participated in the design, and execution. All authors read and approved of the final manuscript.

## Acknowledgements

This work was supported by the American Heart Association (EIA 0740129N) and National Institutes of Health (R01 DK64668).
